# Decreased Equilibrative Nucleoside Transporter 1 (ENT1) Activity Contributes to the High Extracellular Adenosine Levels in Mesenchymal Glioblastoma Stem-Like Cells

**DOI:** 10.3390/cells9081914

**Published:** 2020-08-18

**Authors:** Sebastián Alarcón, María de los Ángeles Toro, Carolina Villarreal, Rómulo Melo, Rodrigo Fernández, Angel Ayuso Sacido, Daniel Uribe, Rody San Martín, Claudia Quezada

**Affiliations:** 1 Laboratorio de Biología Tumoral, Instituto de Bioquímica y Microbiología, Facultad de Ciencias, Universidad Austral de Chile, Valdivia 5090000, Chile; sebastian.alarcon@uach.cl (S.A.); maria.dl.angeles.tb@hotmail.com (M.d.l.Á.T.); carolina.villarrealcabrera@gmail.com (C.V.); daleuri@hotmail.com (D.U.); rodysanmartin@uach.cl (R.S.M.); 2 Servicio de Neurocirugía, Instituto de Neurocirugía Dr. Asenjo, Santiago 7500691, Chile; rmelo@manquehue.net (R.M.); rod.fernandez.g@gmail.com (R.F.); 3 Brain Tumour Laboratory, Fundación Vithas, Grupo Hospitales Vithas, 28043 Madrid, Spain; ayusosacido@gmail.com; 4 Faculty of Experimental Sciences, Universidad Francisco de Vitoria, 28223 Madrid, Spain; 5 Instituto Milenio de Inmunología e Inmunoterapia, Santiago 8320000, Chile

**Keywords:** adenosine, glioblastoma, glioblastoma stem-like cells (GSCs), equilibrative nucleoside transporter 1 (ENT1).

## Abstract

Glioblastoma multiforme is one of the most malignant types of cancer. This is mainly due to a cell subpopulation with an extremely aggressive potential, called glioblastoma stem-like cells (GSCs). These cells produce high levels of extracellular adenosine which has been associated with increased chemoresistance, migration, and invasion in glioblastoma. In this study, we attempted to elucidate the mechanisms that control extracellular adenosine levels in GSC subtypes. By using primary and U87MG-derived GSCs, we associated increased extracellular adenosine with the mesenchymal phenotype. [^3^H]-adenosine uptake occurred mainly through the equilibrative nucleoside transporters (ENTs) in GSCs, but mesenchymal GSCs have lower expression and ENT1-mediated uptake activity than proneural GSCs. By analyzing expression and enzymatic activity, we determined that ecto-5′-nucleotidase (CD73) is predominantly expressed in proneural GSCs, driving AMPase activity. While in mesenchymal GSCs, both CD73 and Prostatic Acid Phosphatase (PAP) contribute to the AMP (adenosine monophosphate) hydrolysis. We did not observe significant differences between the expression of proteins involved in the metabolization of adenosine among the GCSs subtypes. In conclusion, the lower expression and activity of the ENT1 transporter in mesenchymal GSCs contributes to the high level of extracellular adenosine that these GSCs present.

## 1. Introduction

Glioblastoma (GBM) is one of the most malignant types of tumors of the central nervous system [1]. This neoplasia is found in the intracranial tissue/glial cells, which are responsible for supplying functional nutrients and oxygen to the neurons [2]. Despite the variety of modern therapies against GBM, it is still a deadly disease with an extremely poor prognosis [3,4,5]. One reason behind this unsuccessful prognosis is the nature of glioma cells, which infiltrate healthy brain tissue and have a direct impact on the neurologic function of the brain, psychological health, and quality of life, causing serious consequential problems in GBM patients [6,7]. Patients usually have a median survival of approximately 14 to 15 months after diagnosis, despite the current optimized therapies [8]. GBM is characterized by its persistence through self-renewing highly tumorigenic cancer stem-like cells (CSCs) [9]. Evidence suggests that CSCs play an important role during the onset, progression, and recurrence of a tumor and are primarily responsible for radiation and chemotherapy resistance and, therefore, poor patient survival [9,10]. Thus, Glioblastoma Stem-like Cells (GSCs) have emerged as the primary target for therapy against Glioblastoma, but success has been limited [11,12]. Additionally, previous studies have identified two GSC subtypes called proneural and mesenchymal GSCs, which exhibit different metabolic, growth, and malignancy properties and, therefore, different response to therapies [13,14,15]. In culture, mesenchymal GSCs grow as semi-adherent neurospheres, exhibit high glycolytic activity, express markers such as CD44, ALDH1A3, and ITGB5, and give rise to more aggressive tumors than PN GSCs. Proneural GSCs grow as suspension neurospheres, have a high proliferative rate, and express markers such as CD133, SOX2, OLIG2, miR20b, and miR125b [12,13,14,15]. These subtypes may restrain the development of successful GBM therapies unless diagnostic tools are developed and the particular susceptibilities of subpopulations are recognized [16,17,18].

Research performed in recent years shows that the malignant progression of GBM involves several cooperative processes, where adenosine appears to play a fundamental role [6,19,20,21]. Adenosine is an endogenous purine nucleoside that mediates multiple physiological processes in the brain, such as metabolism, cell signaling, purinergic neurotransmission, and inflammation [22,23,24]. This nucleoside is aberrantly increased in the GBM tumor microenvironment. This has been associated with pathogenic adenosine signaling, cell migration/invasion, and chemoresistance in GSCs [6,19,25]. Therefore, knowing and understanding the different cellular mechanisms that modulate extracellular adenosine levels can lead to the development of new and even personalized therapeutic strategies against GSCs.

The extracellular adenosine levels in the tumor microenvironment are the product of different processes. Adenosine may be produced by two ectoenzymes, ecto-5′-nucleotidase (NT5E or CD73) and Prostatic Acid Phosphatase (PAP), that hydrolyze the nucleotide precursor AMP [19,26,27]. Further, adenosine movement through the plasma membrane, mediated by concentrative nucleoside transporters (CNTs) or equilibrative nucleoside transporters (ENTs) [28,29,30], contributes to regulating the extracellular levels of adenosine: since the nucleoside may enter the cells to then be metabolized [31,32,33]. The CNT family is composed of three members (CNT1-CNT3); they actively transport substrates against a chemical gradient in a strict sodium-dependent manner. ENTs are passive transporters specific to eukaryotes, this family is composed of four members (ENT1–4) [30,34]. CNTs and ENTs regulate the physiological cellular uptake of purine and pyrimidine nucleosides and nucleobases, the precursors of nucleotides that are essential for DNA and RNA synthesis [29,30,35]. These two families of transporters are also involved in the uptake of nucleoside analogs currently used in the treatment of solid tumors and other diseases [29,30].

Because nucleoside transporters are important modulators of extracellular adenosine levels and play key roles in the uptake of anti-cancer nucleoside analogs, an important goal for the development of future therapies against GBM is to study and evaluate the expression and/or activity of CNTs and ENTs in GSC derived from human biopsies.

## 2. Materials and Methods

### 2.1. Pharmacological Agents

For in vitro studies NBTI (nitrobenzylthioinosine, Tocris^®^, Bristol, UK) was used as an inhibitor of Equilibrative Nucleoside Transporters and 0.001% DMSO as a vehicle.

### 2.2. Cell Culture

Human U87MG GBM (ATCC^®^, HTB-14^TM^) cell line was grown in differentiation DMEM-F12 medium supplemented with 10% fetal bovine serum and penicillin-streptomycin (Life Technologies, Carlsbad, CA, USA) in standard culture conditions (37 °C and 5% CO_2_).

### 2.3. Glioblastoma Stem-Like Cell Culture

For the generation of GSC from the U87MG cell line (ATCC^®^ HTB-14^TM^), the cells were grown in neurobasal medium (Gibco, Waltham, MA, USA) supplemented with EGF (20 ng/mL; Peprotech©, Rocky Hill, NJ, USA), bFGF (20 ng/mL; Peprotech©), 1X B27 (Gibco™), 1× Glutamax (Gibco) and penicillin/streptomycin (100 U/mL, Gibco) at 37 °C. After 5 days of culture, GSCs were plated to carry out different tests and treatments. Further, GSC primary cultures (PC) were obtained from resected human GBM cultured in M21 medium as described in [36]. Human tissue samples were obtained from patients treated at the hospital HM Universitario Sanchinarro. Permission to use this material was obtained from the ethical review board in Comité Ético de Investigación Clínica del Grupo Hospital de Madrid (ethical code number: 14.10.632-GHM, date of approval: 3 November 2014), and written informed consents were obtained from patients. The work was carried out following the rules of the Declaration of Helsinki. For expansion, GSCs were washed with PBS 1X and then treated for 10 min at 37 °C with StemPro^®^ Accutase^®^ (ThermoFisher, Waltham, MA, USA). Subsequently, the cells were maintained in M21 medium for the generation of new GSCs.

### 2.4. Adenosine Quantification

U87MG and PC GSCs were maintained under standard culture conditions (37 °C, 5% CO_2_) for 4 days. Then, GSCs were washed with PBS 1X two times and incubated in 500 μL of Tyrode’s buffer for 1 h at 37 °C. Later, 200 μL of this incubation medium was mixed with 100 μL of citrate buffer (pH 4). Following derivatization with 2-chloroacetaldehyde (Merck^®^, Darmstadt, Germany) adenosine levels were determined by HPLC fractionation in a Chromolith Performance RP-18e column (Merck^®^) and fluorescent detection [19]. Adenosine concentrations (nM) were normalized to the total protein levels (μg).

### 2.5. Nucleoside Transport Activity

GSCs generated from U87MG and PC were incubated in 200 µL of choline solution (in mM: 5.4 KCl, 1.8 CaCl_2_, 1.2 MgSO_4_, 10 Hepes, 137 choline chloride, pH 7.4) supplemented with 1 µM NBTI or 2 mM hypoxanthine for 30 min. Nucleoside transport activity was assayed in a choline buffer supplemented with 1 µM NBTI or 2 mM hypoxanthine and 10 µM of adenosine containing 2,3[^3^H]-adenosine (2 μCi/nmol)(American Radiolabeled Chemicals, ARC^®^, Inc., St. Louis, MO, USA) for 30 s at 22 °C. Transport was stopped by washing with 1 mL of cold buffer composed of 137 mM choline chloride and 10 mM Tris-Hepes (pH 7.4). GSCs were then centrifuged at 2500× *g* for 5 min at 4 °C and washed again. Then, the pellet was dissolved in 250 µL of 0.5 M HCOOH. Aliquots were sampled for protein determination and radioactivity counting. Particular uptake rates mediated by ENT1 or ENT2 were assigned to transport activities that were inhibited by 1 µM NBTI or 2 mM hypoxanthine, respectively [32]. Total nucleoside uptake in cells mediated by concentrative and equilibrative systems was also measured using a transport buffer containing sodium chloride. Sodium-dependent uptake rates mediated by CNTs were obtained by subtracting adenosine uptake in choline buffer to the total adenosine uptake in buffer containing sodium chloride.

### 2.6. Western Blots

Total proteins extracts (50 μg) obtained from U87MG GSCs and PC GSCs were fractionated by SDS-PAGE, transferred to 0.22 μm PVDF membranes (general electric, GE^®^, Boston, MA, USA) and blocked with 1X PBS/0.05%tween/1%BSA or 5% non-fat milk for 1 h. Then, membranes were incubated overnight with primary antibodies (Table S1) at 4 °C followed by a secondary antibody-HRP conjugate during 1 h. Western blots were revealed using the SuperSignal™ West Dura Extended Duration Substrate kit (Thermo Fisher Scientific) and images were quantified by densitometry analysis (ImageJ, NIH).

### 2.7. RNA Extraction and qRT-PCR

U87MG and PC GSCs were maintained under standard culture conditions (37 °C, 5% CO_2_) for 4 days. Then, total RNA was extracted by using TRIzol Reagent (Thermo Fisher Scientific) and reverse transcription was performed with 1 μg of RNA using the M-MLV Reverse Transcriptase (Thermo Fisher Scientific) following the manufacturer’s instructions. Then, qPCR was performed using the 2^−ΔΔCT^ and ACTB (β-actin) as a normalizer gene using Brilliant II SYBR^®^ Green QPCR Master Mix (#600828, Agilent Technologies, Santa Clara, CA, USA) following the manufacturer’s instructions. The qPCR reactions were performed with 250 nM of each primer (Table S2).

### 2.8. Adenosine Accumulation

U87MG and PC GSCs were maintained under standard culture conditions (37 °C, 5% CO_2_) for 4 days. Then, GSCs were washed with PBS 1X two times and incubated in of M21 medium for 12 h at 37 °C. To assess the effect of blocking equilibrative nucleoside transporters on the accumulation of extracellular adenosine, NBTI was added to the M21 medium for 1 h, at a concentration of 1 µM to inhibit ENT1 and 10 µM to inhibit ENT1 and ENT2 mediated transport, prepared in 0.1% DMSO. Subsequently, adenosine quantification proceeded as described in [19,37]. Adenosine concentrations (nM) were normalized to total protein levels (μg).

### 2.9. CD73 and PAP Activity

PC-GSCs were exposed to 100 μM AMP (Invitrogen, CA, USA) for 30 min in Tyrode buffer pH 6.0 supplemented with erythro-9-(2-hydroxy-3-nonyl) adenine (EHNA) (Tocris, Bristol, UK), and with or without 50 mM α-β-methylene ADP (AOPCP) (Tocris, Bristol, UK). The total incubation medium was immediately centrifuged at 4 °C for 5 min at 2500× *g*. The supernatant was kept on ice. After this procedure, 200 mL of supernatant was mixed with 100 mL of 0.1 M citrate phosphate buffer pH 4.0. Adenosine was quantified using derivatization with 2-chloroacetaldehyde and HPLC with fluorometric detection [37]. The values were expressed as the ratio between generated adenosine to total protein. CD73 activity was the fraction of AMPase activity inhibited by AOPCP. PAP activity was the difference between total AMPase activity and the fraction of AMPase activity inhibited by AOPCP.

### 2.10. Statistics

GraphPad Prism^®^ 6.01 software was used to perform the statistical analysis. Values are shown as mean ± S.D., where n indicates the number of independent experiments. Statistical analysis was carried out on raw data using the Peritz F multiple means comparison test. The Student’s *t*-test was applied for unpaired data. *P* and *p*-adjusted values ≤ 0.05 were considered statistically significant.

## 3. Results

### 3.1. ENTs Contribute to the High Level of Extracellular Adenosine in U87MG GSCs

**Figure 1:**
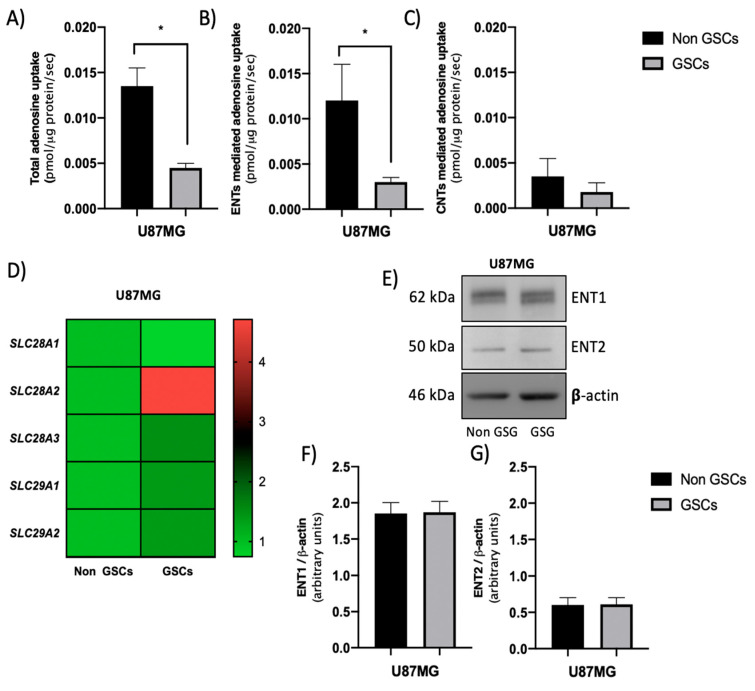
Adenosine transport activity in glioblastoma stem-like cells (GSCs) and non-GSCs. U87MG cells were subjected to different culture conditions to generate GSCs and non-GSCs as described in the Methods section. (**A**) Total adenosine uptake activity mediated by concentrative nucleoside transporter (CNT) and equilibrative nucleoside transporter (ENT) systems were obtained in a transport buffer containing Na^+^. (**B**) ENTs mediated adenosine uptake was determined by using a Na^+^ free buffer. (**C**) The CNT’s component was derived from the difference between total transport activity in Na^+^ containing buffer minus the transport activity in Na^+^ free buffer. (**D**) qRT-PCR of *SLC28A1*, *SLC28A2*, *SLC28A3*, *SLC29A1*, and *SLC29A2* in GSCs and non-GSCs. Values were normalized to ACTB mRNA. (**E**) Representative western blot of ENT1, ENT2, and β-actin in GSCs and non-GSCs. (**F**,**G**) The graphs represent quantification of signals of ENT1 and ENT2 in western blots normalized against β-actin signals. The plots represent the means ± S.D. *, *p* < 0.05. *n* = 5.

**Figure 2:**
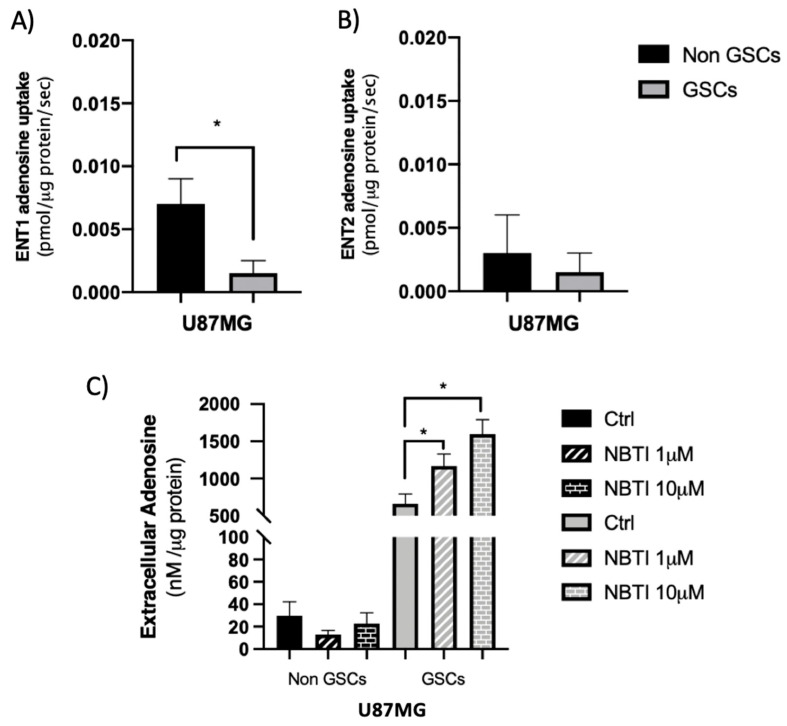
GSCs exhibit lower ENT1 transport activity relative to non-GSCs. U87MG cells were subjected to different culture conditions to generate GSCs and non-GSCs as described in the Methods section. (**A**) The graph represents ENT1 mediated adenosine uptake as the fraction of transport in a Na^+^ free buffer inhibited by 1 μM NBTI. (**B**) The plot represents ENT2 mediated adenosine uptake as the fraction of transport in a Na^+^ free buffer inhibited by 2 mM hypoxanthine. (**C**) Extracellular adenosine levels were quantified in culture medium exposed to 1 µM NBTI (to inhibit ENT1) or 10 µM NBTI (to inhibit ENT1 and ENT2) as described in Methods 2.8. The plots represent the means ± S.D. *, *p* < 0.05. *n* = 5.

GSCs present higher extracellular adenosine levels than non-GSC differentiated GBM cells [19]. To determine the contribution of concentrative nucleoside transporters (CNTs) and equilibrative nucleoside transporter (ENTs) activity in the regulation of extracellular adenosine levels, we performed adenosine uptake assays as described in Methods 2.5. For this, we used GSCs derived from a U87MG cell line as our model and compared them with U87MG cells in differentiating conditions. As seen in Figure 1A, U87MG-derived GSCs adenosine uptake was less than a third of non-GSCs. Subsequently, the contribution of CNTs or ENTs in this context was evaluated. We observed that adenosine uptake in non-GSCs is mainly mediated by ENTs, however, GSCs significantly decreased ENT-mediated adenosine transport relative to non-GSCs (Figure 1B). Due to decreased ENTs activity, the uptake of this nucleoside becomes mediated in a similar proportion by CNTs and ENTs in GSCs (Figure 1B,C). When evaluating adenosine uptake mediated by CNTs, we observed that there are no significant differences between GSCs and non-GCSs (Figure 1C). Subsequently, transcript levels for the genes coding for CNT1 (*SLC28A1*), CNT2 (*SLC28A2*) and CNT3 (*SLC28A3*), and ENT1 (*SLC29A1*) and ENT2 (*SLC29A2*) in GSCs and non-GCS were evaluated by qRT-PCR. Regarding genes coding for CNTs, only a significant increase in the transcript level of the *SLC28A2* gene was observed in GSCs compared to non-GSCs (Figure 1D). For genes encoding ENTs, we did not observe significant variations in transcript levels between GSCs and non-GSCs (Figure 1D). Because we observed a significant difference in ENT-mediated adenosine uptake between GSCs and non-GSCs, we proceeded to assess the expression and activity levels of the major members of this transporter family. Western blot analysis of ENT1 and ENT2 proteins (Figure 1E) shows that there are no significant differences in ENT1 (Figure 1F) and ENT2 (Figure 1G) protein levels between GSCs and non-GSCs. Subsequently, we performed tests to elucidate the contribution of the ENT1 and ENT2 transporters on the extracellular uptake and accumulation of adenosine in GSCs and non-GSCs. We observed a significant decrease in ENT1 mediated adenosine uptake in GSCs compared to non-GSCs (Figure 2A). No variations were observed in ENT2 mediated uptake under the conditions described above (Figure 2B). Finally, we developed adenosine accumulation assays as described in Methods 2.8. It was previously reported that GSCs accumulate more extracellular adenosine than non-GSC [19] and as seen in Figure 2C, after inhibition of ENT1 mediated uptake, by using NBTI 1 μM, in GSCs, extracellular adenosine levels significantly increased compared to the control. When inhibiting transport by ENT1 and ENT2, by using NBTI 10 μM, we observed an increase in extracellular adenosine levels only compared to the control, without significant changes in the content of extracellular adenosine when ENT1 mediated transport alone is inhibited.

Based on the evidence above, we conclude that lower ENT1 transport activity contributes to higher extracellular adenosine levels in U87MG-derived GSCs.

### 3.2. GSCs Subtypes Exhibit Differential Expression and Activity Levels of ENTs Which Correlate with Their Extracellular Adenosine Levels

The existence of multiple cellular subpopulations of cancer cells has been previously described. In particular, two main subtypes of patient-derived GSCs can be easily distinguished based on their molecular signature, therapy responses, and patient survival. The best-accepted classification includes the mesenchymal (MES) and proneural (PN) subtypes [13,14,15].

**Figure 3:**
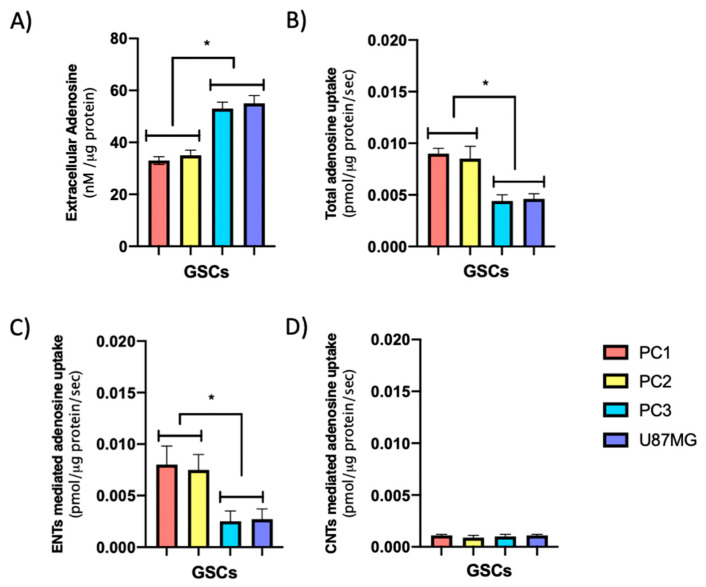
Adenosine uptake activity in proneural (PN) and mesenchymal (MES) GSCs. PN GSCs (PC1 and PC2) and MES GSCs (PC3 and U87MG) were previously characterized (Supplementary Figure S1). (**A**) Extracellular adenosine levels (nM) in GSC subtypes were quantified as described in Methods 2.4. (**B**) Total adenosine uptake mediated by concentrative (CNTs) and equilibrative (ENTs) systems were obtained in a transport buffer containing Na^+^. (**C**) ENTs adenosine mediated uptake was determined by using a Na^+^ free buffer. (**D**) CNTs component was derived from the difference between total transport activity in the Na^+^ containing buffer minus the transport activity in the Na^+^ free buffer. The plots represent the means ± S.D. *, *p* < 0.05. *n* = 5.

For this study, we used three primary cultures of GSCs (PC1, PC2, and PC3) previously characterized according to their growth and malignancy properties [36]. We have observed that the PC1 and PC2 GSCs grow as neurospheres in suspension, express PN markers such as CD133, OLIG2, SOX2, miR20b, and miR125b, and have lower glycolytic activity than the PC3 and U87MG GSCs. In contrast, the PC3 GSCs grows as semi-adherent neurospheres, and like the U87MG GSCs, they have a high glycolytic activity, have high expression of CD44 and ALDH1A3 mesenchymal markers and low expression of miR20b and miR125b with respect to the PC1 and PC2 GSCs (see Figure S1). Taken together, these results suggest that the PC1 and PC2 GSCs exhibit a PN signal, while the PC3 and U87MG GSCs show MES signal. We evaluated if there are differences in extracellular adenosine levels based on the phenotypic landscape of the GSCs. There were two significantly different groups for extracellular adenosine levels (Figure 3A). We observed that the GSCs corresponding to PC1 and PC2, with PN phenotype, presented lower extracellular adenosine levels compared to the GSCs generated from PC3 and U87MG, exhibiting MES markers. Subsequently, we evaluated adenosine transport activity in the aforementioned GSCs. We observed that MES PC3 and U87MG GSCs uptake less adenosine than PN PC1 and PC2 GSCs (Figure 3B), which corresponds with the higher extracellular adenosine levels exhibited by MES GSCs compared to PN GSCs (Figure 3A). Subsequently, the influence of CNTs or ENTs in this context was evaluated. We observed that adenosine uptake for both GSCs subtypes is mainly mediated by ENTs (Figure 3C,D). The ENT-mediated uptake of adenosine is higher in PN PC1 and PC2 GSCs compared to MES PC3 and U87MG GSCs (Figure 3C). We also observed that only a minimal fraction of adenosine uptake is mediated by CNTs in the studied GSCs (Figure 3D).

**Figure 4:**
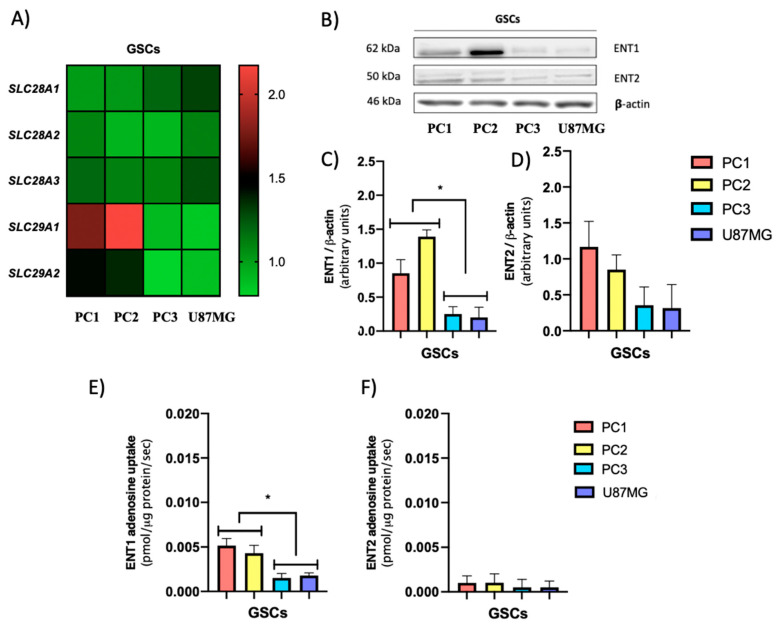
PN GSCs exhibit higher levels of ENT1 expression and activity than MES GSCs. (**A**) Relative transcript levels from *SLC28A1*, *SLC28A2*, *SLC28A3*, *SLC29A1*, and *SLC29A2* genes in PN (PC1 and PC2) and MES (PC3 and U87MG) GSCs. Values were normalized to ACTB mRNA expression. (**B**) Representative western blot of ENT1, ENT2, and β-actin in GSCs subtypes. (**C**,**D**) The graphs depict Western blot quantification for ENT1 and ENT2 signals normalized against β-actin signals. (**E**,**F**) The graphs represent ENT1 and ENT2 mediated adenosine uptake as the fraction of the transport in a Na+ free buffer inhibited by 1 μM NBTI or 2 mM hypoxanthine, respectively. The plots represent the means ± S.D. *, *p* < 0.05. *n* = 5.

Subsequently, the transcript levels of genes coding for CNTs and ENTs in the studied GSCs were evaluated by qRT-PCR as described in Methods 2.7. There were no significant differences in the transcript levels of the *SLC28A1*, *SLC28A2*, and *SLC28A3*, coding for CNTs, between the different GSCs studied (Figure 4A). Regarding genes encoding ENTs, the group of PN GSCs, PC1 and PC2, presented a higher level of gene transcript for *SLC29A1* and *SLC29A2* compared to the group of MES GSCs.

Then, we evaluated the expression and activity of the members of the equilibrative nucleoside transporter family described above. By western blot analysis (Figure 4B), we observed that GSCs PC1 and PC2 present higher ENT1 levels than the GSCs PC3 and U87MG groups. Furthermore, there were no significant differences in ENT2 protein levels between the different GSCs studied (Figure 4D).

Finally, to elucidate the contribution of ENT1 and ENT2 transporters to extracellular adenosine accumulation in the groups of GSCs described above, adenosine transport assays were performed. For ENT1, PC1, and PC2 (PN) adenosine uptake was significantly higher than in PC3 and U87MG (MES) (Figure 4E). For ENT2, we did not observe variations in adenosine transport activity among the GSCs (Figure 4F).

Based on this evidence, we can conclude that in MES GSCs the lower expression and activity of ENT1 correlates with higher extracellular adenosine levels compared to PN GSCs.

### 3.3. The GSC Subtypes Differentially Express Adenosine Producing Enzymes But Not Adenosine Metabolizing Enzymes

**Figure 5:**
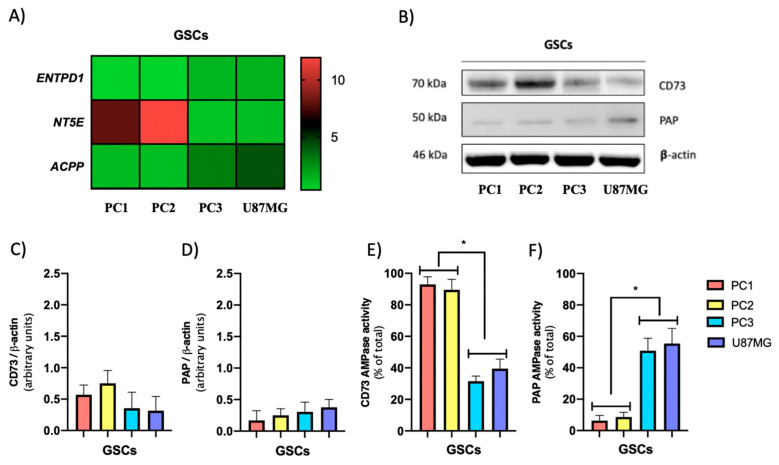
Expression and activity of AMP metabolizing enzymes in proneural and mesenchymal GSCs. (**A**) Relative transcript levels from ENTPD1, NT5E, and ACPP genes in proneural (PC1 and PC2) and mesenchymal (PC3 and U87MG) GSCs. Values were normalized to ACTB mRNA expression. (**B**) Representative western blot of CD73, PAP, and β-actin in GSC subtypes. (**C**,**D**) Western Blot quantification of CD73 and PAP signals normalized against β-actin signals. (**E**,**F**) CD73 and PAP-mediated AMPase activity evaluated in proneural and mesenchymal GSCs. The graphs represent the means ± S.D. *, *p* < 0.05. *n* = 5.

Another mechanism that can modulate extracellular adenosine levels is its production through adenine nucleotides metabolization, a process driven by the action of ectoenzymes that produce and hydrolyze AMP. Based on this, the transcript level of genes coding ecto-nucleoside triphosphate diphosphohydrolase (E-NTPDase), ecto-5′-nucleotidase (CD73), and prostatic acid phosphatase (PAP) were evaluated by qRT-PCR. We observed that the PN GSCs (PC1 and PC2) exhibit significantly higher transcript levels of the *NT5E* gene coding for the CD73 protein compared to the MES GSCs (PC3 and U87MG) (Figure 5A). Regarding the ACPP gene, which encodes PAP, we observed higher transcription levels in MES GSCs compared to PN GSCs (Figure 5A). No significant changes in ENTPD1 transcript levels (Figure 5A), which codes for the E-NTPDase, were observed among the different GSCs studied. Based on this information, the levels of CD73 and PAP proteins in the GSC subtypes were analyzed by western blot (Figure 5B).

**Figure 6:**
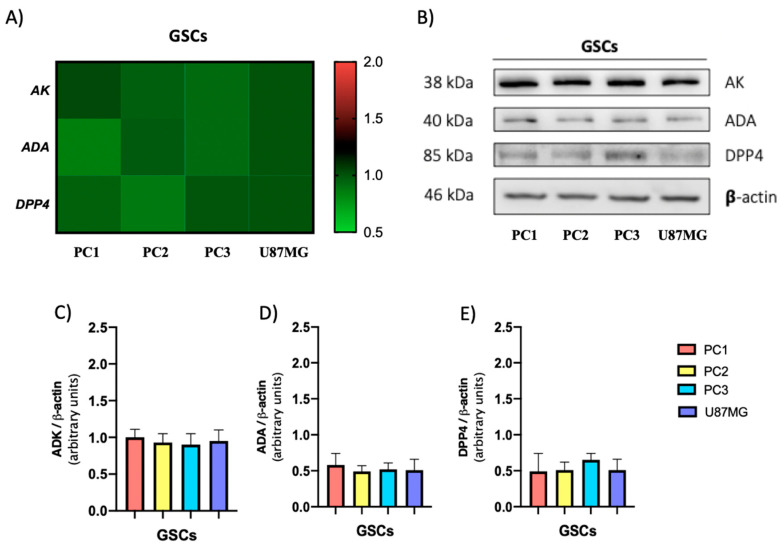
Expression of enzymes related to adenosine metabolism in proneural and mesenchymal GSCs. (**A**) Relative transcript levels from AK, ADA, and DPP4 genes in proneural (PC1 and PC2) and mesenchymal (PC3 and U87MG) GSCs. Values were normalized to ACTB mRNA expression. (**B**) Representative western blot of AK, ADA, DPP4, and β-actin in subtypes of GSCs. (**C**–**E**) Western Blot quantification of AK, ADA and DPP4 signals normalized against β-actin signals. The plots represent the means ± S.D.

We observed that CD73 is expressed in both types of GSCs, but mostly in PN GSCs (Figure 5C). PAP was expressed predominantly in MES GSCs (Figure 5D). Subsequently, the activity of CD73 and PAP proteins was evaluated as described in Methods 2.9. We observed that in PN GSCs much of the AMPase activity is driven by CD73 (Figure 5E), whereas for the MES GSCs subtype, we observed that AMPase activity presented a mixed component between CD73 and PAP (Figure 5D,E). Finally, another mechanism that can modulate extracellular adenosine levels in GSCs is its degradation. Therefore, by qRT-PCR we evaluated the transcript levels of the gene coding for adenosine deaminase (ADA), an enzyme that degrades adenosine to inosine, and the transcript levels of the gene coding for DPP4, a protein that serves as a binding protein for extracellular ADA in humans. No significant variations in the transcript levels of the genes coding for ADA and DPP4 were observed between the GSCs described above (Figure 6A). Further, as shown in Figure 6D,E there are no significant variations in the level of ADA and DPP4 proteins between PN and MESGSCs. Therefore, we conclude that adenosine degradation is not the preponderant factor in modulating the differential extracellular adenosine levels observed in the GSCs studied.

## 4. Discussion

The therapy resistance and relapse presented by the glioblastoma (GBM) are driven by glioma stem cells (GSCs) [38]. In recent years GSCs have been categorized based on their molecular and phenotypic differences in mesenchymal (MES) GSCs that have higher rates of proliferation in vitro and are more resistant to radiation than proneural (PN) GSCs [10,16]. Primary PN GBM, originally responsive to treatment, may relapse as MES tumors which become refractory to treatment [38]. Thus, understanding the properties of both GSC subpopulations is clinically relevant for the management of patients. Our research group previously demonstrated that GSCs have high levels of extracellular adenosine compared to non-GSCs [19], which is associated with pathogenic signaling through the adenosine receptor which in turn mediates the greater chemoresistant and invasive potential observed in GBM [6,19,39]. Although adenosine-producing enzymes in glioblastoma cells were previously identified [27], there is no information regarding the contribution of nucleoside transporters nor ectoenzymes to extracellular adenosine levels in GSCs subtypes. Nucleoside transporters play a key role in the physiologic control of adenosine by regulating the extracellular adenosine available for cell-surface receptors [40]. Information regarding the expression of CNTs and ENTs is also scarce; however, in 2006, an article indicated that in U87 cells, uptake of radiolabeled adenosine appeared to be via both an ENT Na^+^-independent and a Na^+^-dependent CNT mechanism, which is in line with our results [40]. Regarding total transport, we observed a drastic reduction in the uptake of adenosine in GSCs compared to non-GSCs, which leads to an increase in the extracellular level of this nucleoside. Alterations in the expression levels of nucleoside transporters lead to increased extracellular adenosine levels in various cell types [32,33,41]. Despite this, we observed no significant variations in the transcript or protein levels of ENT1 or ENT2 transporters between non-GSCs and GSCs (Figure 1B,E–G). The mechanisms involved in regulating the expression and activity of CNTs and ENTs have not yet been fully described. However, studies show the dual distribution of ENT1 and ENT2 between the intracellular compartment and the plasma membrane [42,43]. Furthermore, Alarcon et al., 2017, showed that changes in transporter Vmax may be due to altered transport efficiency, the number of transporters at the plasma membrane, or both [32]. Additionally, phosphorylation of ENT1 by CK2 leads to a reduction in Vmax and this decrease in the membrane transporter affects its capacity to transport adenosine [44]. ENT1 activity may be modulated by oligomerization with ENT2 [45]; however, more studies are required to determine how the transition towards GSCs of U87MG can affect ENT1 activity. A different situation was observed when comparing PN and MES GSCs, where ENT1 expression is affected. Some studies indicate that ENT1 expression can be regulated by hypoxia [46,47]; therefore, differences in hypoxic factor activation between PN and MES GSCs may influence adenosine uptake [48,49,50].

Due to the relatively high activity of intracellular adenosine kinase and the normally low intracellular adenosine levels, the net flux through ENTs is inwardly directed under normal conditions [51], such that the request for ENT activity is reflected in the permanence of extracellular adenosine. Several reports in diverse cell types agree with our obtained data. Studies using Human Umbilical Vein Endothelial Cells (HUVECs) and Placenta Microvascular Endothelial Cells (PMECs) have shown that control of extracellular adenosine levels via P1 receptors also involves ENT modulation, in particular, the ENT1 and ENT2 subtypes [52,53]. A study in samples from patients with schizophrenia shows that the inhibition of ENT1 transport activity leads to elevated adenosine levels [54]. Additionally, the ENT1 antagonist mediated a dose-dependent increase in extracellular adenosine levels, activating the different adenosine receptors [55]. This has also been described in animal models, where intrastriatal administration of ENT1 inhibitors increased extracellular adenosine levels in the striatum of R6/2 mice [56]. The ENT1-null mice also have increased adenosine plasma levels [57,58].

In the cells evaluated, CNT-mediated adenosine transport corresponds to a smaller fraction of adenosine transport in both GSC subtypes. Based on the Km values of CNTs compared to ENTs (CNT2 8 µM, CNT3 15 µM, ENT1 40 µM, ENT2 140 µM) [59], it seems that CNTs are key players in ensuring the availability of precursors for the synthesis of nucleotides through the rescue pathway. However, from a pharmacological point of view, increased ENT activity in GSCs offers an opportunity for the development of an anti-tumor treatment directed at GSCs. Nucleoside analogues such as 5-fluorouracil could be efficiently taken up by the PN GSCs. Currently, no clinical studies have demonstrated the susceptibility of GBM to this family of drugs.

Interestingly, we noted that in PN GSCs most AMPase activity is supported by CD73. However, in MES GSCs, we detected that AMPase activity is co-conducted by CD73 and PAP. Previous studies have demonstrated a correlation between high CD73 levels with the enhanced migratory and invasive capacity of glioma cells [6,60]. Further, we recently demonstrated that PAP is involved in adenosine-dependent EMT marker expression, migration, and GSCs invasion, characteristics that have been attributed to the MES subtype [6,13,14,15]. Based on their Km values (PAP: 0.37–2 mM: CD73: 24 µM) using AMP as a substrate [61,62,63], a preponderant role of CD73 could be conjectured around the formation of adenosine over PAP. Further, it has been suggested that the conversion of AMP to adenosine may be produced by CD73 and, less efficiently, by alkaline phosphatase (PAP) [64]. However, the acidification of the tumor microenvironment caused by lactic acid which is secreted from cancer cells could lead to disturbances around the activity of the proteins in question [65,66,67]. Studies suggest that PAP acts as an ecto-5′-nucleotidase with relative selectivity for AMP at a neutral pH and as a generic ectonucleotidase with selectivity for AMP, ADP, and ATP at an acidic pH [68]. This indicates a possible advantage in the formation of adenosine in mesenchymal GSCs containing PAP (Figure 5E,F). Concerning to extracellular adenosine catabolism, it has been described that DPP4 serves as a binding protein for extracellular ADA in humans, anchoring it to the cell surface and thus reducing local levels of adenosine [69]. On the other hand, Ado can be degraded by the action of the ADA enzyme intra and extracellularly thus, changes in its expression or activity can lead to variations in the capacity of the enzyme to degrade adenosine [70,71,72]. We did not observe significant differences in the expression of ADA and DPP4 between the studied GSC subtypes. Further, we were unable to detect changes in ADA activity in the studied GSC subtypes (data not shown). Therefore, we did not find a correlation between altered adenosine catabolism and the high extracellular levels present in GSCs. Although lower ENT1 activity in mesenchymal GSCs may have a preponderant role in the accumulation of extracellular adenosine, another very important factor that can influence this condition is the different metabolic properties between the GSC subtypes and the ability to produce and extrude nucleotides. In this study, we showed that MES GSCs also exhibit higher extracellular levels of ATP, ADP, and AMP than those observed in the PN cells (data not shown). Based on this, we deduced that the extracellular metabolism of nucleotides in the MES subtype is displaced towards the synthesis of adenosine from precursors released by these same cells.

## 5. Conclusions

The extracellular concentration of adenosine is tightly regulated by multiple mechanisms that lead to transient or sustained adenosine accumulation. This study evaluated three important pathways that can regulate the amount of extracellular adenosine, these being the catabolism of precursor nucleotides, the nucleosidases degradative pathway, and the flux of adenosine through the plasma membrane. Based on the obtained results, we can conclude that MES GSCs have a higher level of extracellular adenosine than PN GSCs, with the lower expression and activity of the ENT1 transporter, being the most relevant engine in limiting adenosine uptake to be cleared by the cells. Finally, these results highlight adenosinergic signaling as a potential marker and therapeutic target of the most aggressive phenotype of GSCs.

## Supplementary Materials

The following are available online at https://www.mdpi.com/2073-4409/9/8/1914/s1, Table S1: List of antibodies used in this study; Table S2: List of primer sequences used for qRT-PCR; Figure S1: Characterization of the GSC subtypes; Supplementary Materials and Methods S1: microRNA Isolation and RT-qPCR, RNA extraction and RT-qPCR, Glycolysis Cell-Based Assay.
