# Supplementary material for: Decreased Equilibrative Nucleoside Transporter 1 (ENT1) Activity Contributes to the High Extracellular Adenosine Levels in Mesenchymal Glioblastoma Stem-Like Cells
Source: Cells. 2020 Aug 18;9(8):1914. doi: 10.3390/cells9081914 (PMC7463503; doi:10.3390/cells9081914)
Supplement: Supplementary file 1 [file cells-09-01914-s001.zip › Supplementary/Primary Antibody Table S1.docx]

**Table S1.** List of antibodies used in this study.

| **Target** | **Antibody** | **Company** |
| --- | --- | --- |
| ENT1 | 11337-1-AP | Proteintech^TM^ |
| ENT2 | NBP1-69312 | Novus Biologicals |
| CD73 | D7F9A | Cell Signaling |
| PAP | D3Y5P | Cell Signaling |
| AK | sc-514588 | Santa Cruz Biotechnology |
| ADA | sc-53152 | Santa Cruz Biotechnology |
| DPP4 | D6D8K | Cell Signaling |
