# Supplementary material for: Decreased Equilibrative Nucleoside Transporter 1 (ENT1) Activity Contributes to the High Extracellular Adenosine Levels in Mesenchymal Glioblastoma Stem-Like Cells
Source: Cells. 2020 Aug 18;9(8):1914. doi: 10.3390/cells9081914 (PMC7463503; doi:10.3390/cells9081914)
Supplement: Supplementary file 1 [file cells-09-01914-s001.zip › Supplementary/Characterization of the different subtypes of GSCs.docx]

#
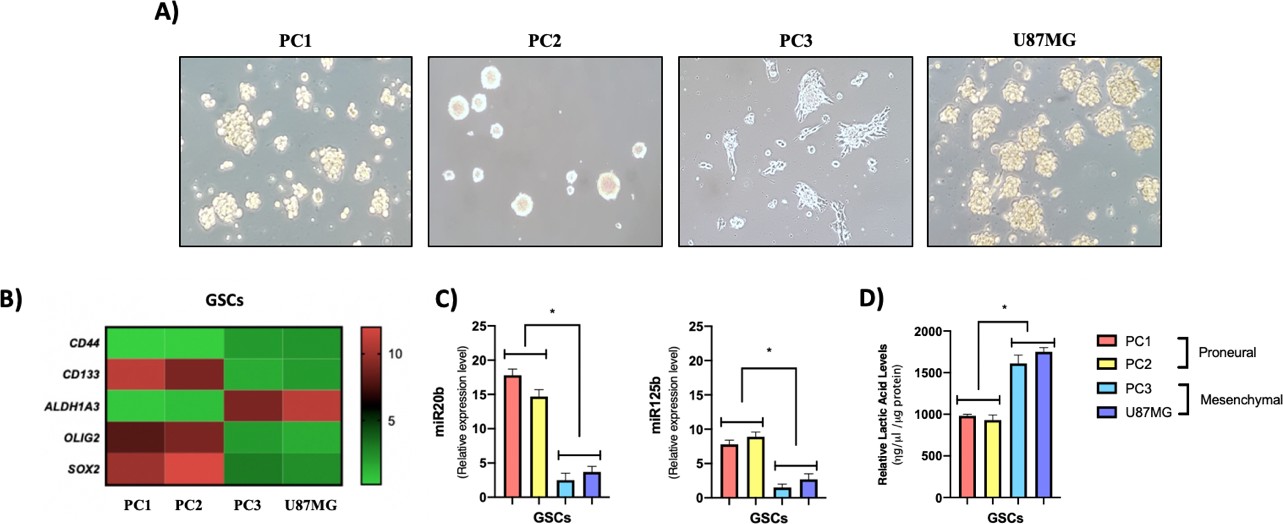


**Figure S1: Characterization of proneural GSCs and mesenchymal GSCs.** These structures were generated and maintained as described in methods. Proneural GSCs (PC1 and PC2), Mesenchymal GSCs (PC3 and U87MG). **A)** Light microscopy images of neurospheres (GSCs) obtained from primary cultures (PC1, PC2 and PC3) and the U87MG cell line. **B)** RT-qPCR of CD44, CD133, ALDH1A3, OLIG2, and SOX2 in different GSCs subtypes. Values were normalized to ACTB mRNA expression. **C)** RT-qPCR of miR-20b and miR-125b. Values were normalized to miR-RNU48 expression. **D)** Elevated glycolytic activity in Mes GSCs compared with PN GSCs. The plots represent the means ± S.D. *P < 0.05. n = 5.

# Supplementary Materials and Methods S1:

**microRNA Isolation and RT-qPCR**

U87MG- and PC- GSCs were maintained under standard culture conditions (37ºC, 5% CO_2_) during 4 days. Then, microRNAs were extracted using a miRNA isolation kit (mirVanaTM, Thermo Fisher Scientific). microRNA samples were quantitated using the Qubit HS RNA kit (Thermo Fisher Scientific) with a Qubit 3.0 Fluorometer (Thermo Fisher Scientific), following manufacturer’s

instructions. Reverse transcription was performed with 200 ng of RNA using the High Capacity cDNA Reverse Transcription Kit (Applied Biosystems) following the manufacturer's instructions. qPCR was performed using the 2ˆ(–delta delta CT) and mirRNU48 as a normalizer gene using Brilliant II SYBR® Green QPCR Master Mix (#600828, Agilent Technologies) following the manufacturer’s instructions. qPCR reaction was performed with 250 nM of each primer (Table S2).

# RNA extraction and RT-qPCR

U87MG- and PC- GSCs were maintained under standard culture conditions (37ºC, 5% CO_2_) during 4 days. Then total RNA was extracted by TRIzol Reagent (Thermo Fisher Scientific) and reverse transcription was performed with 1 µg of RNA using the M-MLV Reverse Transcriptase (Thermo Fisher Scientific) following the manufacturer's instructions. qPCR was performed using the 2ˆ(–delta delta CT) and ACTB (β-actin) as a normalizer gene using Brilliant II SYBR® Green QPCR Master Mix (#600828, Agilent Technologies) following the manufacturer’s instructions. qPCR reaction was performed with 250 nM of each primer (Table S2).

# Glycolysis Cell-Based Assay.

Briefly, GSCs were seeded in a 96-well plate at a density of 1×104 cells per well in 120 µL of neurosphere medium and cultured overnight in a CO_2_ incubator at 37 °C. Then, glycolysis cell-based assay kit (Lactate Assay Kit, Sigma-Aldrich) was used to measure the glycolytic activity in GSC samples according to the manufacturer’s instructions. Lactate concentrations (ng/μl) were normalized to the total protein levels (µg).
